# Luminescent copper(i) coordination clusters: structure–property relationships, transformation into hybrid functional materials, and emerging optoelectronic applications

**DOI:** 10.1039/d6ra07011f

**Published:** 2026-10-12

**Authors:** L. Anjo, N. Ananikian, G. Amatuni, V. Avagyan, M. Ivanyan, G. Davoodi

**Affiliations:** a Advanced Materials and Microelectronics Laboratory, CANDLE Synchrotron Research Institute 31 Acharyan Yerevan 0022 Armenia anjo@asls.candle.am; b Soil and Water Resources Engineering, Armenian National Agrarian University Yerevan 0025 Armenia; c Alikhanyan National Scientific Laboratory Yerevan 0036 Armenia

## Abstract

Atomically precise luminescent copper(i) coordination clusters have emerged as a sustainable, earth-abundant, and structurally versatile class of materials, offering a compelling alternative to noble-metal (Au, Ag) luminophores. This review comprehensively addresses recent advances spanning the period 2020 to 2026 in the rational design, photophysical characterization, and functional hybridization of these clusters. We systematically discuss nuclearity control (Cu_2_ to Cu_22_), ligand engineering strategies, and the structure–property relationships that govern emission color and quantum yield, with particular emphasis on Cu⋯Cu metallophilic interactions, tetrahedral distortion parameter (Δ*θ*), and ligand rigidity. The roles of metal to ligand charge transfer (MLCT), ligand to metal charge transfer (LMCT), cluster-centered (CC) transitions, and thermally activated delayed fluorescence (TADF) are critically analyzed. Sonochemical synthesis is evaluated as a potentially useful laboratory-scale approach for accelerating nucleation and facilitating *in situ* hybridization; however, its scalability, passivation efficiency and ability to suppress aggregation-caused quenching remain to be demonstrated through matched comparisons with conventional routes. The review further explores the transformation of discrete Cu(i) clusters into advanced hybrid functional materials incorporating polymer matrices, metal–organic frameworks (MOFs), oxide supports, and carbon-based scaffolds. Emerging applications in chemical sensing, TADF-based optoelectronics (*Φ*_PL_ up to 99.9%, EQE >35%), and visible-light photocatalysis are critically evaluated. Challenges including Cu(i) oxidation susceptibility, ACQ, and synthetic scalability are addressed, and future directions including defect engineering, machine-learning-assisted design, and sonochemical hybridization are proposed.

## Abstract 2

An evidence-based overview links the structures and excited-state photophysics of luminescent Cu(i) coordination clusters with hybrid materials and emerging sensing, optoelectronic and photocatalytic applications.

## Graphical abstract

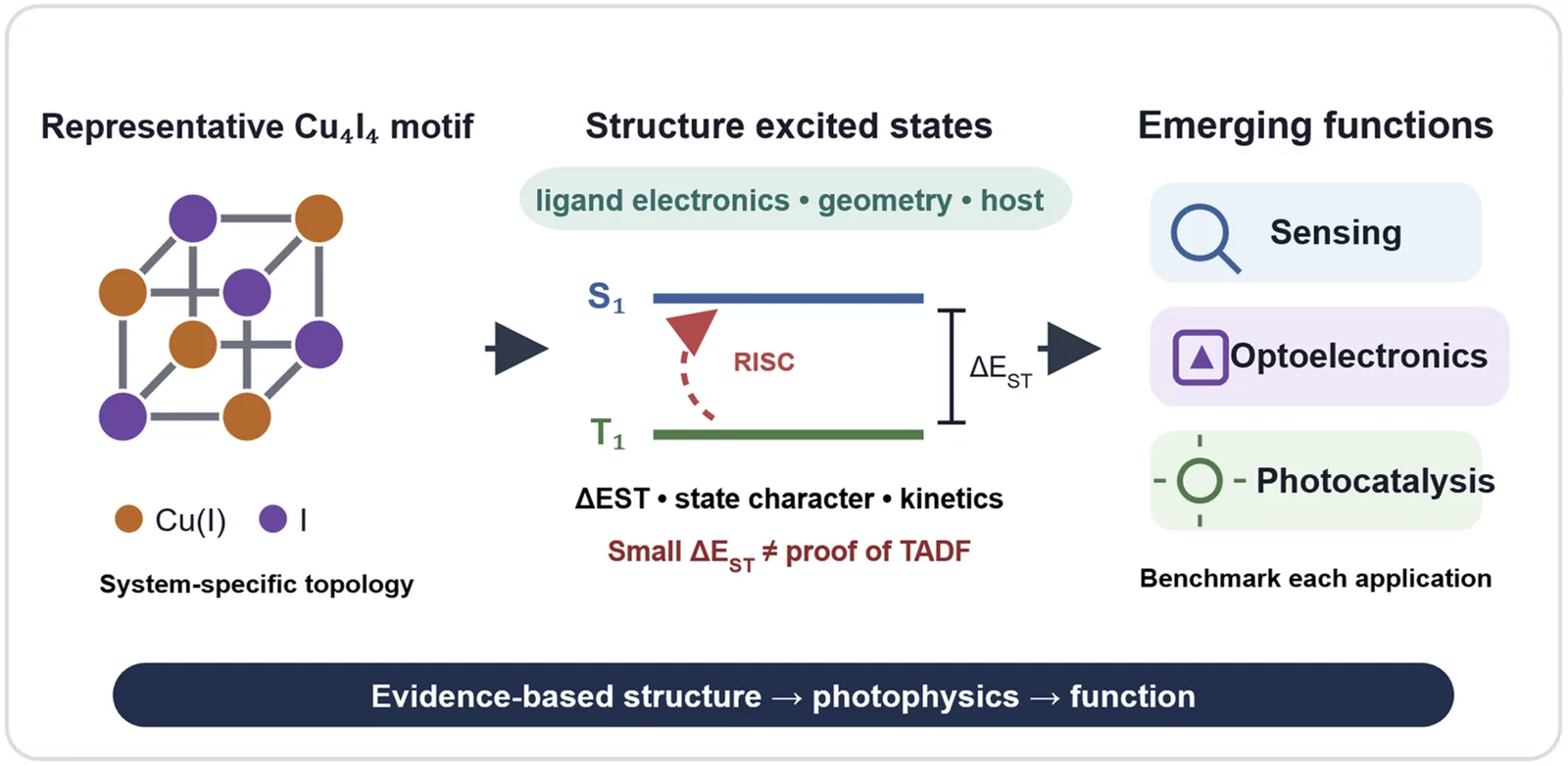

## 1. Introduction

Atomically precise coordination clusters of coinage metals represent a frontier at the interface of molecular chemistry and nanoscale materials science.^1,2,62,65,75,76^ Unlike plasmonic nanoparticles, these clusters possess well defined stoichiometries and crystal structures, enabling direct correlations between atomic architecture and macroscopic optical properties. Among them, luminescent gold and silver clusters have historically dominated the literature, owing to their chemical stability and well-established synthetic protocols.^5,6^ However, the substantial costs associated with these noble metals, combined with their limited structural diversity relative to their copper analogues, have stimulated intense interest in earth abundant alternatives. Copper(i) coordination clusters are particularly compelling candidates. Copper is the third most abundant transition metal in the Earth's crust, and Cu(i) (d^10^) centers support a rich diversity of coordination geometries from linear to tetrahedral and cluster nuclearities ranging from dinuclear (Cu_2_) to high-nuclearity hydride-bridged species (Cu_22_).^7,23^ The photoluminescence of Cu(i) clusters arises from a combination of metal to ligand charge transfer (MLCT), ligand to metal charge transfer (LMCT), halide to ligand charge transfer (XLCT), cluster centered (CC) transitions, and Cu⋯Cu cuprophilic interactions that are central to thermally activated delayed fluorescence (TADF).^10–12^ These photophysical characteristics, combined with potentially near-unity exciton utilization, position Cu(i) clusters as candidates for the next generation of energy efficient lighting and display technologies.^13,32^ Despite these merits, the field faces significant challenges. Cu(i) is susceptible to oxidation to the photophysically silent Cu(ii) state, particularly under ambient conditions. Aggregation caused quenching (ACQ) limits performance in the solid state, and scalable synthetic routes beyond small scale solvothermal methods remain underdeveloped.^16,17^ Reviews of luminescent metal nanoclusters remain dominated by Au and Ag systems, while coverage of discrete Cu(i) coordination clusters particularly those exploring the interplay of nuclearity, ligand design, sonochemical synthesis, and hybridization is confined to short perspectives and focused mini-reviews.^7,8^ The present review aims to fill this gap by providing a critical, data-driven analysis of luminescent Cu(i) coordination clusters. It is organized as follows: Section 2 addresses rational design principles, including nuclearity control, ligand strategies, and synthetic routes, with sonochemical methods highlighted for their scalability and green credentials. Section 3 discusses photophysical properties and emission mechanisms. Section 4 suggests crystallographically grounded structure property relationships, supported by quantitative comparisons of Δ*θ*, Cu⋯Cu distances, and HOMO–LUMO gaps. Section 5 describes the transformation of discrete clusters into hybrid functional materials. Section 6 surveys emerging applications, and Section 7 outlines current challenges and future directions. This review reflects the expertise of our group in sonochemistry, oxide materials, and photoluminescence spectroscopy.^18,19,21,41^ To provide a clear overview, the comprehensive journey of these emissive materials spanning from green synthetic pathways to structural optimization and device integration is schematically encapsulated in the Graphical Abstract. Specifically, this review deciphers how alternative eco-friendly strategies (such as sonochemical and mechanochemical methods) govern the assembly of discrete tetranuclear copper(i) scaffolds. Furthermore, we establish the fundamental structure property relationships that dictate the singlet triplet energy splitting Δ*E*_ST_ and accelerate the reverse intersystem crossing (RISC) process. Finally, recent advances in embedding these robust clusters into polymeric matrices for the fabrication of hybrid functional materials and thin-film organic light-emitting diodes (OLEDs) are thoroughly discussed (Fig. 1).

**Fig. 1:**
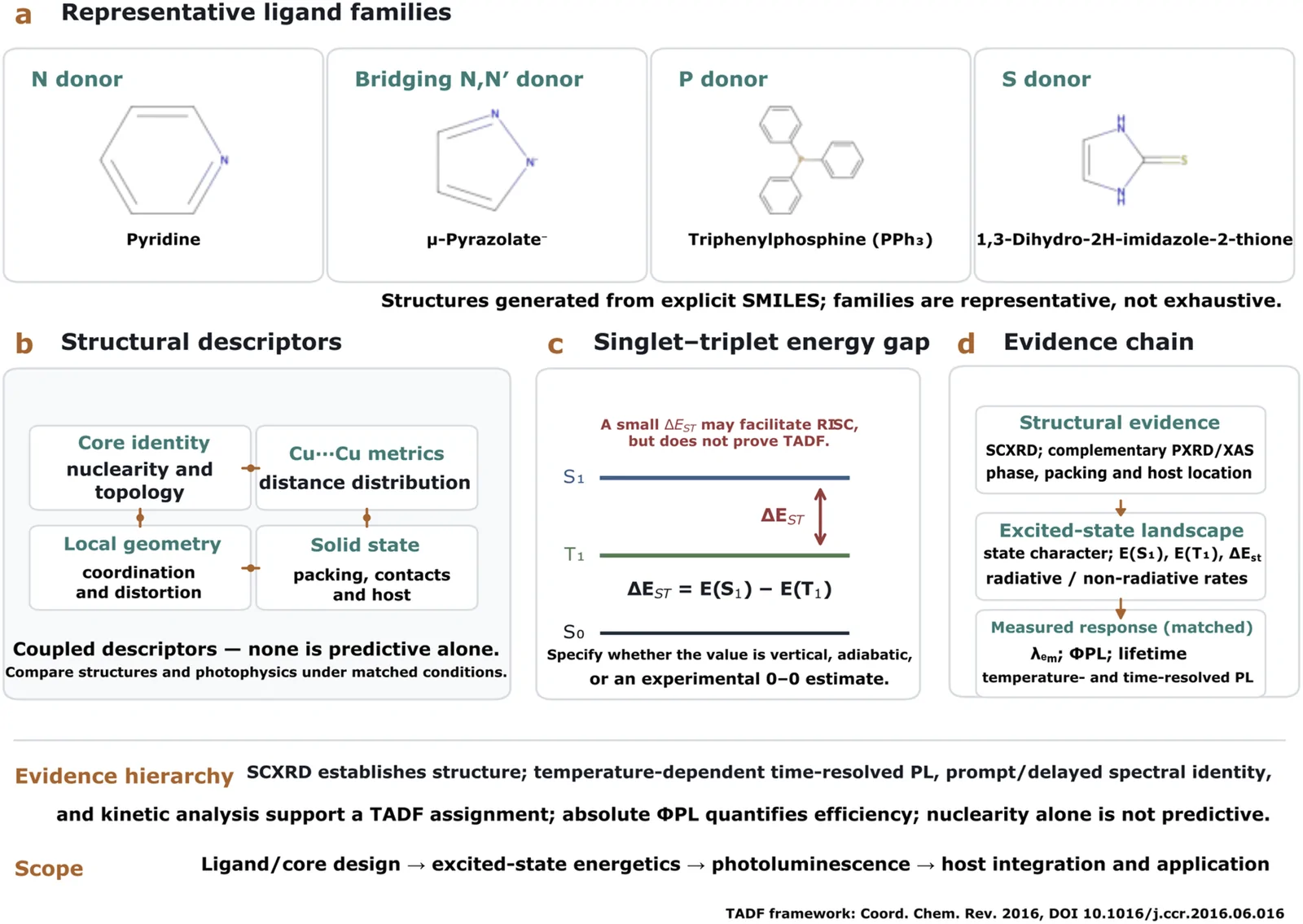
Evidence-based framework for luminescent Cu(i) coordination clusters: (a) representative ligand families, (b) key structural descriptors, (c) definition and method dependence of Δ*E*_ST_, and (d) the structure-to-photophysics evidence chain. A small Δ*E*_ST_ may facilitate RISC but does not alone establish TADF.

## 2. Design and synthesis of luminescent Cu(i) coordination clusters

The rational design of luminescent Cu(i) coordination clusters requires simultaneous control over nuclearity, ligand coordination environment, Cu⋯Cu interactions, and synthetic conditions. Three principal design variables nuclearity, ligand selection, and synthetic route are discussed below.

### 2.1 Nuclearity control

Nuclearity defines the size and connectivity of the Cu(i) core and can influence orbital delocalization, vibrational freedom, metal–halide interactions, and the number of accessible excited states. However, nuclearity is not an independent or universally predictive descriptor of *λ*_em_ or *Φ*_PL_ because changes in nuclearity are usually accompanied by changes in ligand identity, halide composition, core topology, crystal packing, and measurement conditions. Pyrazolate ligands commonly support cyclic trinuclear Cu_3_ assemblies, whereas chalcogenone and phosphine ligands can stabilize Cu_2_–Cu_5_ and higher-nuclearity cores.^4,24,55^ High-nuclearity hydride clusters, including Cu_20_H_13_ and Cu_22_H_19_ species supported by N-containing triphosphine ligands, extend this structural range.^22,23^ However, their photophysical properties have not been characterized under conditions directly comparable with those used for low-nuclearity emitters. Consequently, apparent changes in *λ*_em_ or *Φ*_PL_ across different nuclearities should be treated as family specific observations rather than universal trends.

### 2.2 Ligand strategies

Ligands perform three simultaneous functions in Cu(i) clusters: they protect Cu(i) from oxidation, define the cluster geometry, and modulate photophysical output through electronic and steric effects. The principal ligand classes are summarized below:

• N-donor ligands (pyridyl, pyrazolyl, imidazolyl, triazolyl):^56^ these afford both σ-donation and π-acceptance, enabling neutral or cationic clusters with strong stimuli-responsiveness. Imidazole-2-thione/selone derivatives stabilize Cu_2_–Cu_5_ cores; the chalcogenone identity (S *vs.* Se) directly controls emission color and intensity through differential electronic communication.^26^

• Phosphine-based ligands: tripodal NP_3_ ligands stabilize high nuclearity hydride clusters (Cu_20_, Cu_22_) and enforce rigid coordination geometries that suppress excited-state flattening.^23,59^ Phenoxazine-modified diphosphines invert excited state ordering (see Section 3), enabling near-unity exciton utilization.^10^

• Pyrazolate and triazole based ligands: these support cyclic trinuclear Cu_3_ assemblies with strong cuprophilic stacking. Halogen-bonded (XB) networks in multi bromo pyrazolate clusters generate excimeric states with negative thermal quenching (NTQ), maintaining *Φ*_PL_ > 90% at elevated temperatures;^4^ related mechanochromic and vapochromic dinuclear and cubane-type Cu(i) halide systems reported by independent groups further substantiate this structure-switching behavior.^68–71^

• Chalcogen and halide co-ligands: Br/I-rich environments favor halogen bonded excimeric networks. Iodide co ligands enhance spin orbit coupling and promote TADF by reducing the singlet–triplet splitting (Δ*E*_ST_).^35^

Ligand rigidity is a particularly decisive parameter: rigid π-conjugated frameworks minimize nonradiative decay through suppression of excited state geometric distortion, while chiral ligands enable circularly polarized luminescence (*C*_PL_).^13,38^

### 2.3 Synthetic routes

#### 2.3.1 Conventional solvothermal synthesis

Traditional solvothermal methods remain prevalent for producing high-quality single crystals suitable for X-ray diffraction analysis. CuX (X = Cl, Br, I), Cu_2_O, or Cu(NO_3_)_2_ precursors are reacted with ligands under elevated temperature in mixed solvent systems (*e.g.*, acetonitrile/DMF). One pot reaction affords neutral clusters *via in situ* reduction or ligand deprotonation,^4,26^ and related CuI secondary building units have also been assembled into extended one-dimensional luminescent coordination polymers.^28^

#### 2.3.2 Mechanochemical synthesis

A recent study demonstrated a solvent-free mechanochemical route to zero-dimensional Cu(i)-based organic–inorganic halide superclusters through dynamic ligand fusion. The isolated products exhibited high solid-state photoluminescence quantum yields, although their performance cannot be attributed solely to mechanochemical processing because the two products differ in composition and nuclearity. Accordingly, the study demonstrates synthetic feasibility and reduced solvent use, but does not by itself establish general scalability or a universal suppression of aggregation-caused quenching.^25^

#### 2.3.3 Sonochemical synthesis

Sonochemical synthesis exploits acoustic cavitation the formation, growth and implosive collapse of bubbles under ultrasonic irradiation to generate transient localized high-temperature and high-pressure conditions.^17,53^ These effects can accelerate mass transport, nucleation and interfacial mixing in materials synthesis. In copper-containing systems, cavitation-generated reactive species may also participate in precursor reduction, although the resulting oxidation state and coordination structure must be verified experimentally. Sonochemical processing may therefore offer shorter reaction times and facilitate one-pot integration with oxide, carbon or polymer hosts. However, the available literature does not yet provide matched Cu(i)-cluster studies demonstrating universally narrower size distributions, superior passivation, reduced ACQ or industrial scalability relative to solvothermal and mechanochemical routes. These potential advantages should consequently be regarded as hypotheses requiring quantitative benchmarking of yield, phase purity, energy consumption, *Φ*_PL_ retention, reproducibility and scale.

Table 1 compares selected Cu(i) systems for which directly traceable synthetic and photophysical data are available. Quantitative cross-comparison remains limited because the reported studies differ in composition, nuclearity, reaction scale, purification protocol, physical state and photophysical measurement conditions. Consequently, the available evidence demonstrates the feasibility of alternative synthetic routes but does not establish universal improvements in yield, scalability or emission efficiency relative to conventional solution synthesis.

**Table 1:** Evidence-based comparison of conventional, mechanochemical and sonochemical routes for selected luminescent Cu(i) systems. SCXRD = single-crystal X-ray diffraction; PXRD = powder X-ray diffraction; ACQ = aggregation-caused quenching

Route	Representative system	Structural evidence	Verified photophysical result	Critical interpretation	Ref.
Solution crystallization	P,N-supported Cu_4_I_4_ clusters 1–5	SCXRD	Compounds 3 and 4: ≈460 nm; compounds 1, 2 and 5: ≈460 nm plus a ≈570 nm component	A ligand-matched series supporting substituent-dependent ^3^XLCT/^3^CC state contributions; not a synthesis-route comparison	27
Solution synthesis	Chalcogenone-supported Cu(i) clusters 1 and 3	SCXRD and spectroscopy	*Φ*_PL_ = 19 and 11%; *τ* = 21.55 and 6.57 µs, respectively	Values belong to two distinct compounds and must not be interpreted as a universal nuclearity trend	24
Solution synthesis	Functionalized copper(i) phenylthiolate clusters	SCXRD and optical spectroscopy	Visible emission varies with core geometry, Cu⋯Cu separation and thiolate substituent	Supports conditional structure–emission relationships within the reported family; quantitative route superiority was not tested	20
Solution synthesis	[DBFDP]_2_Cu_4_I_4_, [DCzDBFDP]_2_Cu_4_I_4_ and [DtBCzDBFDP]_2_Cu_4_I_4_	SCXRD and temperature/time-resolved spectroscopy	*λ*_em_/*Φ*_PL_: ≈620 nm/5%, 491 nm/46% and 480 nm/65%, respectively	A controlled ligand series demonstrating suppression of low-lying ^3^CC emission by donor substitution	88
Mechanochemical synthesis	0D Cu(i)-based luminescent superclusters formed through dynamic ligand fusion	PXRD and spectroscopic characterization	Stimuli-responsive dual-mode emission reported	Demonstrates solvent-free synthetic feasibility, but does not establish universal scalability, superior passivation or reduced ACQ	25
Sonochemical synthesis	No directly matched, structurally authenticated Cu(i)-cluster series identified	Cluster-specific comparative SCXRD/PXRD dataset unavailable	Directly comparable *λ*_em_, *Φ*_PL_ and *τ* data are unavailable	Claimed advantages remain hypotheses until yield, phase purity, energy use, reproducibility and scale are benchmarked against conventional routes	17,53

### 2.4 Critical assessment of synthetic routes

Despite the potential green-chemistry advantages of mechanochemical and sonochemical methods, both routes currently trade off single-crystal quality against throughput: solvothermal synthesis remains the method of choice whenever unambiguous SCXRD structural assignment is required, whereas mechanochemical and sonochemical products are more frequently characterized by powder XRD and spectroscopy alone, leaving their precise Cu⋯Cu geometries and Δ*θ* values less rigorously established. Batch-to-batch reproducibility of mechanochemical superclusters, in particular, has not yet been benchmarked across independent laboratories, and standardized protocols for scaling sonochemical synthesis beyond gram quantities remain to be reported. Closing this gap is a prerequisite for translating the green-chemistry advantages highlighted above into industrially relevant production. Reported sonochemical protocols to date have been demonstrated only at sub-100 mg to gram scale,^17,19,21^ and quantitative throughput or cost comparisons against solvothermal or mechanochemical routes have not yet been reported for any Cu(i) cluster system; claims of superior scalability, passivation, and ACQ suppression for sonochemical and mechanochemical methods should therefore presently be regarded as promising at laboratory scale rather than industrially established.

## 3. Photophysical properties and emission mechanisms

The photophysical behavior of luminescent Cu(i) coordination clusters arise from a complex interplay of electronic transitions, structural rigidity, and intermolecular interactions. Unlike noble-metal cluster systems, Cu(i) clusters benefit from the d^10^ electronic configuration, which suppresses spin forbidden metal-centered (MC) quenching transitions while permitting efficient charge-transfer and cluster-centered emission pathways.^10,11^

### 3.1 Emission mechanisms

The primary emission pathways in Cu(i) clusters encompass: (i) metal-to-ligand charge transfer (MLCT), involving Cu(i) d → ligand π* excitation; (ii) ligand to metal charge transfer (LMCT); (iii) halide-to-ligand charge transfer (XLCT); and (iv) cluster-centered (CC) transitions arising from delocalized metal–metal bonding orbitals.^12,34,35^ In most emissive Cu(i) clusters, the lowest-lying singlet (S_1_) and triplet (T_1_) states exhibit (M + X)LCT character, with small singlet triplet energy gaps (Δ*E*_ST_ typically 0.1–0.2 eV),^10,12^ consistent with the foundational photophysical framework for Cu(i)-based TADF and triplet-harvesting emitters.^50,51^ This small Δ*E*_ST_ enables efficient thermally activated reverse intersystem crossing (RISC), proceeding from T_1_ → S_1_, at room temperature the hallmark of TADF.^11,32^ Unambiguous assignment of TADF requires variable-temperature, time-resolved emission spectroscopy demonstrating a temperature-activated prompt/delayed emission ratio and lifetime shortening, as established for archetypal Cu(i) TADF emitters.^50–52^ For many of the clusters discussed in this review, TADF character is instead inferred indirectly from a small Stokes shift, a microsecond-scale lifetime, and/or a TD-DFT-predicted Δ*E*_st_, and should be regarded as tentative until confirmed by such temperature-dependent measurements; throughout Sections 3 and 4, we distinguish, where the primary literature permits, between clusters with experimentally confirmed TADF and those for which a TADF mechanism is proposed on the basis of steady-state photophysics or theory alone. Note that RISC necessarily runs uphill in energy from T_1_ to S_1_, enabled by thermal activation; this is distinct from standard intersystem crossing (ISC), which proceeds in the energetically downhill S_1_ → T_1_ direction. A particularly important recent discovery is the phenomenon of excited-state reversal in Cu_4_I_4_ clusters bearing electron-donating phenoxazine (PXZ) diphosphine ligands, where ligand-centered (LC) states are pushed below the non-radiative MC states of the Cu_4_I_4_ core,^10^ complementing other Cu_4_I_4_ cubane systems in which ligand modification (*e.g.*, aryl isocyanide substitution) similarly tunes dual-emission behavior and mechanical-stimulus sensitivity.^54^ This state inversion enables near-unity exciton utilization through balanced TADF (52%) and phosphorescence (48%), achieving *Φ*_PL_ approaching 98% and external quantum efficiencies (EQE) exceeding 35% in prototype electroluminescent devices,^57^ a benchmark now approached by several independently reported Cu(i)-based and Cu-doped noble-metal-cluster OLEDs.^79,80^ The halide identity (Cl < Br < I) systematically modulates the emission mechanism: softer I^−^ ligands enhance spin orbit coupling, promote RISC, and red shift emission, while rigid N- or P-donor ligands suppress geometric distortion in the excited state, reducing non-radiative decay.^35,37^ Excitation-dependent emission has also been reported in dual-bridged Cu(i) dimers with unusually long Cu⋯Cu distances (>6 Å), where positional isomerism of pyridylphosphine ligands switches the dominant transition from pure TADF to mixed MLCT/XLCT character.^33^

### 3.2 Factors influencing photophysical properties

Four structural and environmental parameters are primarily responsible for variation in *λ*_em_, *Φ*_PL_, and excited-state lifetime (*τ*):

• Nuclearity and Cu⋯Cu interactions: nuclearity and Cu⋯Cu separation can influence cluster-centered orbital energies and the accessibility of emissive states. Nevertheless, neither parameter exhibits a universal monotonic relationship with *λ*_em_ or *Φ*_PL_ across chemically unrelated clusters. Their effects are most reliably evaluated within ligand-matched, polymorph-matched, or otherwise structurally controlled series.^20,24,25^

• Ligand rigidity and π-conjugation: rigid frameworks (*e.g.*, tripodal NP_3_, chalcogenone derivatives, XB-modified pyrazolates) minimize excited state flattening, yielding microsecond-scale lifetimes (10–60 µs) and high radiative rate constants (*k*_r_). Flexible ligands promote solvent- and temperature dependent quenching.^4,26^

• Packing and intermolecular interactions: halogen bonding (XB) in pyrazolate clusters generates excimeric states with negative thermal quenching (NTQ). Crystal packing in hybrid systems can additionally suppress ACQ.^4^

• External stimuli: thermochromism, mechanochromism and solvatochromism can arise from reversible changes in Cu⋯Cu separation, coordination geometry, crystal packing or ligand conformation. The direction and magnitude of the response depend on the particular compound and cannot be generalized across different Cu(i) cluster families.^47,49,69,70,82^

Representative *Φ*_PL_ values span 11–99.9% in the solid state, with *τ* ranging from 1 to 60 µs, demonstrating performance comparable to or exceeding that of many Au/Ag analogues when Cu(i) oxidation is controlled.^10,33^ A ligand-substituent-controlled structure–emission relationship has been reported within a family of P,N-supported Cu_4_I_4_ clusters. These compounds retain a rare octahedral-type Cu_4_I_4_ topology comprising a Cu_4_ parallelogram capped by two µ_4_-iodides and bridged by two µ_2_-iodides. Compounds 3 and 4 exhibit a single band near 460 nm assigned to a triplet halide-to-ligand charge-transfer (^3^XLCT) state, whereas compounds 1, 2 and 5 additionally exhibit a lower-energy component near 570 nm assigned to a triplet cluster-centred (^3^CC) state.^27^ These observations demonstrate ligand-dependent control of the relative emissive-state contributions but do not independently establish TADF (Fig. 2).

**Fig. 2:**
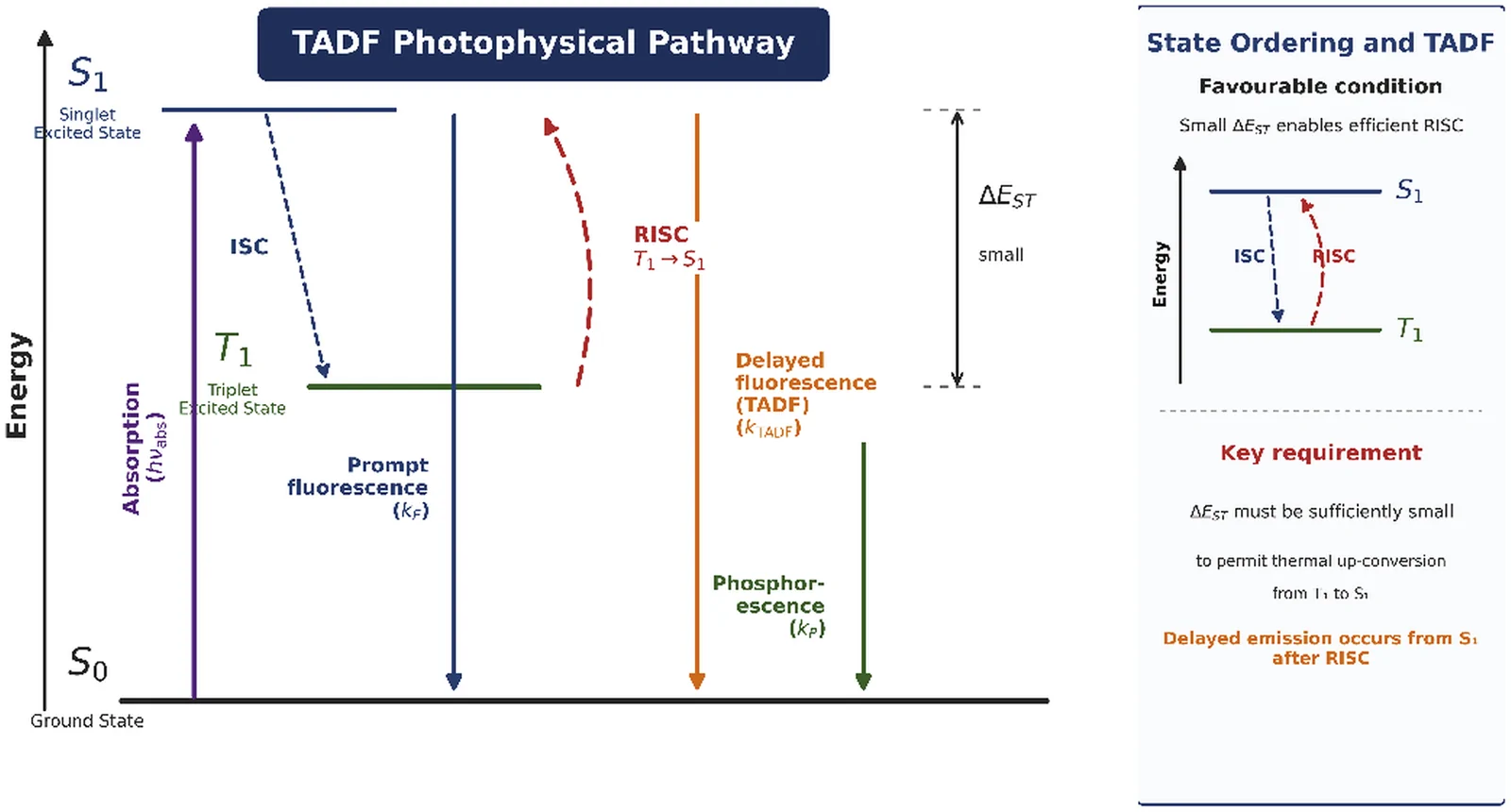
Generalized TADF mechanism in Cu(i) luminophores. Excitation populates S_1_, followed by prompt fluorescence or ISC to T_1_. Thermal activation may enable RISC from T_1_ to S_1_ when Δ*E*_st_ is sufficiently small and spin–vibronic coupling is favorable, producing delayed fluorescence. Phosphorescence and non-radiative relaxation remain competing pathways.

### 3.3 Critical assessment of photophysical assignments

A number of the *Φ*_PL_ values compiled above are reported without accompanying temperature-dependent lifetime measurements, which are required to unambiguously distinguish TADF from simple phosphorescence or from a mixture of both pathways; in their absence, mechanistic assignments in the primary literature are sometimes inferred rather than directly demonstrated, a challenge also emphasized in systematic studies explicitly designed to disentangle phosphorescence from TADF *via* singlet triplet splitting control.^52^ Comparability across studies is further limited by inconsistent reporting conventions (absolute *vs.* relative *Φ*_PL_, solid-state *vs.* solution measurements, and varying excitation wavelengths). Standardized, temperature and time-resolved photophysical characterization protocols, following recently proposed best-practice frameworks for metal nanocluster photophysics,^77^ would substantially strengthen the structure–property correlations discussed throughout this review.

## 4. Structure property relationships and crystallographic insights

The establishment of quantitative structure property correlations is central to the rational design of high-performance Cu(i) luminophores. Single crystal X-ray diffraction (SCXRD) provides the primary structural metrics Cu⋯Cu distances, coordination bond angles, and intermolecular contacts that can be directly correlated with photophysical outcomes. While copper(i) coordination clusters exhibit distinct closed-shell d^10^ photophysical configurations governed by metallophilic Cu⋯Cu interactions, valuable geometric and structural insights can be drawn from open-shell tetranuclear and trinuclear copper systems as purely geometric and topological analogues. It must be emphasized that Cu(i) clusters possess a closed-shell d^10^ configuration with no unpaired valence electrons; therefore, ground-state antiferromagnetic exchange coupling constants (*J*) and spin-state configurations applicable to open-shell Cu(ii) systems do not exist for Cu(i). The references to open-shell Cu(ii) frameworks here serve exclusively as geometric topology maps (*e.g.*, spatial connectivity, vibrational mode analysis) and are not intended as direct electronic or magnetic analogues. For instance, the theoretical modeling of multinuclear copper networks, including tetramer, square, and diamond-chain scaffolds, provides frameworks for mapping spatial connectivity and quantum coherence pathways.^39,45^ Studies by Ananikian *et al.* on thermal entanglement and cyclic four-spin exchange parameters in Cu(ii) and Ni(ii) complexes illustrate how subtle geometric alterations affect electronic configurations.^39,45^ Adapting these structural topology frameworks to d^10^ systems allows for a deeper rationalization of excited state configurations, particularly when evaluating structural distortions Δ*θ* and non-radiative relaxation parameters in highly symmetric cubane like Cu_4_ cluster cores.

### 4.1 Nuclearity *versus* emission characteristics

Nuclearity determines the number and connectivity of Cu(i) centers and can therefore influence cluster-centered orbital delocalization, metal–halide interactions, vibrational degrees of freedom, and the density of accessible excited states. Nevertheless, the available evidence does not support a universal relationship between nuclearity and either the emission wavelength or photoluminescence quantum yield. Comparisons across Cu_2_, Cu_3_, Cu_4_, and higher-nuclearity clusters usually involve simultaneous changes in ligand identity, halide composition, coordination geometry, crystal packing, and measurement conditions. Nuclearity is therefore a structural classification parameter rather than an independent predictor of photophysical behavior. The data compiled in Table 2 illustrate this limitation. The dinuclear complex [Cu_2_I_2_(DPO)_2_] emits at 432 nm, whereas the Cu_4_I_4_ compounds span approximately 480–620 nm and exhibit *Φ*_PL_ values ranging from approximately 5 to 90%.^10,33,88^ This large variation among isonuclear Cu_4_I_4_ clusters is incompatible with a simple nuclearity-controlled model. Instead, it reflects differences in ligand donor strength, excited-state character, structural reorganization, and sample environment. For example, donor-functionalization of the DBFDP ligand changes the balance between ligand-involved charge-transfer and cluster-centered states without changing the Cu_4_I_4_ nuclearity, producing substantial variations in both *λ*_em_ and *Φ*_PL_.^88^ Similarly, [PXZDBFDP]_2_Cu_4_I_4_ and [PhPXZDBFDP]_2_Cu_4_I_4_ possess the same nominal Cu_4_I_4_ core but differ markedly in excited-state ordering and emission efficiency.^10^

**Table 2:** Selected structure–photophysical data for luminescent Cu(i) coordination clusters. DPO = bis(2-diphenylphosphinophenyl)ether; DBFDP = 2,9-bis(diphenylphosphino)dibenzofuran; CT = charge transfer; M/XLCT = metal/halide-to-ligand charge transfer; CC = cluster-centred; LCT = ligand-centred; PH = phosphorescence; NR = not reported comparably

Cluster	Structural feature	Cu⋯Cu (Å)	*λ*_em_ (nm)	*Φ*_PL_ (%)	Δ*E*_ST_ (eV)	Photophysical assignment	Ref.
[Cu_2_I_2_(DPO)_2_]	I-bridged Cu_2_ core; no significant cuprophilic interaction	3.38	432	NR	0.108	M/XLCT; TADF supported by temperature-dependent measurements and TD-DFT	33
[DBFDP]_2_Cu_4_I_4_	Phosphine-chelated Cu_4_I_4_ cube	NR	∼620^a^	5	0.16	Mixed CT/^3^CC emission	88
[DCzDBFDP]_2_Cu_4_I_4_	Donor-modified rigid Cu_4_I_4_ cube	NR	491	46	0.07	Predominantly CT-based TADF (>90%)	88
[DtBCzDBFDP]_2_Cu_4_I_4_	^3^CC-suppressed Cu_4_I_4_ cube	NR	480	65	NR	Ligand-involved CT emission; TADF/PH competition	88
[PXZDBFDP]_2_Cu_4_I_4_	Excited-state-reversed Cu_4_I_4_ cube	NR	508^b^	∼90^b^	0.04^b^	LCT; 52% TADF and 48% PH at ambient temperature	10
[PhPXZDBFDP]_2_Cu_4_I_4_	Cu_4_I_4_ cube retaining low-lying CC states	NR	498^b^	∼11^b^	0.02^b^	Predominantly PH (>99%); inefficient TADF despite small Δ*E*_ST_	10

a Low-energy CC component of broad dual emission.

b Measured in a 20 wt% cluster-doped BCPO film; values across rows were obtained under different conditions.

High-nuclearity hydride clusters such as Cu_20_H_13_ and Cu_22_H_19_ further demonstrate the difficulty of cross-family comparisons. These compounds were primarily developed and characterized in the context of hydride coordination and catalysis rather than through standardized photophysical measurements.^22^ Their electronic structures, ligand environments, and metal–hydride bonding differ fundamentally from those of phosphine-stabilized Cu_4_I_4_ emitters. Consequently, their broad or red-shifted emission, where reported, cannot be used to establish a general nuclearity-dependent bathochromic shift. Nuclearity may become mechanistically informative when examined within a ligand-matched series in which the metal-to-ligand ratio, halide, packing environment, and measurement protocol are controlled. In the absence of such controls, apparent nuclearity emission correlations should be treated as qualitative observations rather than transferable design rules. Fig. 3a therefore presents the individual literature values without connecting lines or fitted trends. Accordingly, the available data do not support a universal nuclearity-dependent red shift or a monotonic decrease in *Φ*_PL_ (Fig. 3a).

**Fig. 3:**
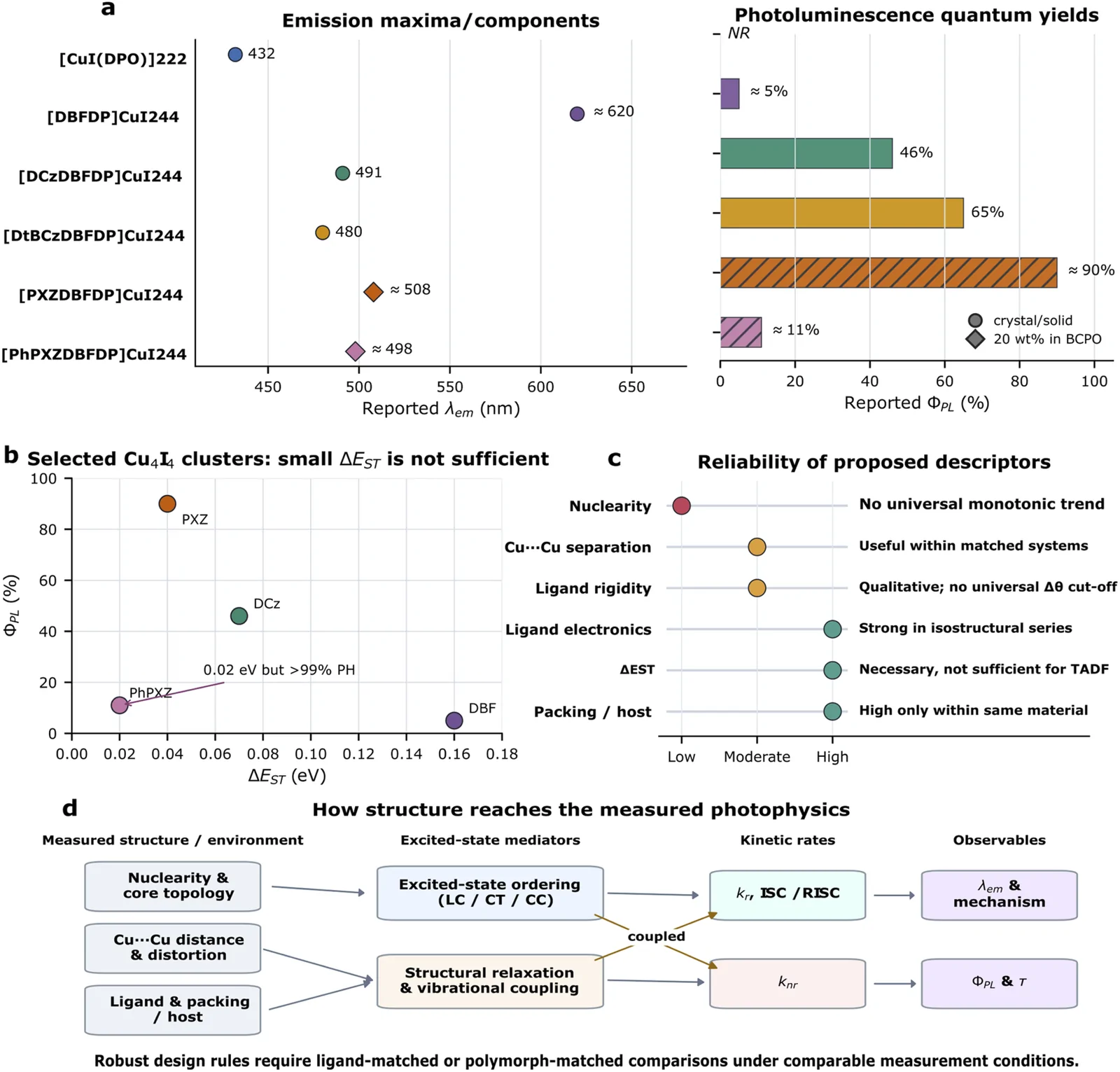
Critical structure–photophysical relationships in Cu(i) clusters: (a) reported *λ*_em_ and *Φ*_PL_, (b) Δ*E*_ST_–*Φ*_PL_ relationship, (c) reliability of structural descriptors, and (d) coupled structural and excited-state effects on photoluminescence.

### 4.2 Ligand rigidity, electronic structure, and excited-state reorganization

Structural rigidity can improve the emission efficiency of Cu(i) compounds by limiting the large-amplitude excited-state distortion commonly associated with partial Cu(i)-to-Cu(ii)-like electronic reorganization. Reduced structural relaxation can decrease non-radiative decay; however, rigidity should not be represented by a universal tetrahedral-distortion threshold. Parameters derived from ground-state crystal structures describe only the crystallographic geometry and do not directly measure the magnitude of excited-state reorganization. Moreover, the numerical value of a distortion parameter depends on its definition, the selected coordination angles, and the coordination number of the Cu(i) center. Accordingly, claims such as Δ*θ* < 10° invariably producing high *Φ*_PL_, or Δ*θ* > 25° necessarily causing quenching, are not supported by the heterogeneous dataset considered here. The relevant photophysical relationships are more accurately expressed through the radiative and non-radiative rate constants:
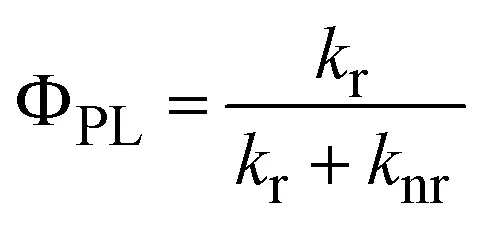

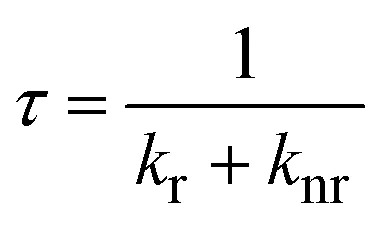


A rigid environment may reduce *k*_nr_, but *Φ*_PL_ and *τ* also depend on *k*_r_, excited-state energy, spin–orbit coupling, and the accessibility of competing states. A high radiative rate does not, by itself, imply a long emission lifetime. Multistate Cu(i) systems containing thermally coupled singlet and triplet states require a more detailed kinetic description. Ligand electronics provide a particularly clear example of a structure property relationship that can be evaluated within closely related Cu_4_I_4_ clusters. In the DBFDP series, donor substitution progressively reduces the contribution of low-lying, weakly radiative cluster-centered states and increases ligand-involved charge-transfer character. [DBFDP]_2_Cu_4_I_4_ exhibits a low-energy cluster-centered component near 620 nm and *Φ*_PL_ of approximately 5%, whereas the donor-modified [DCzDBFDP]_2_Cu_4_I_4_ and [DtBCzDBFDP]_2_Cu_4_I_4_ emit at 491 and 480 nm with *Φ*_PL_ values of 46% and 65%, respectively.^88^ Because the nuclearity and general Cu_4_I_4_ topology are retained, this series provides stronger evidence for ligand-controlled excited-state tuning than comparisons among unrelated nuclearities. The PXZ/PhPXZ pair provides an additional and particularly informative comparison. In [PXZDBFDP]_2_Cu_4_I_4_, strong donor acceptor coupling stabilizes ligand-centered charge-transfer states below the less radiative cluster-centered states. In a 20 wt% BCPO film, this compound exhibits *λ*_em_ = 508 nm, *Φ*_PL_ ≈ 90%, and an ambient emission contribution of approximately 52% TADF and 48% phosphorescence.^10^ The related [PhPXZDBFDP]_2_Cu_4_I_4_ retains low-lying cluster-centred character and shows *Φ*_PL_ of only approximately 11% under the same nominal host loading, despite having a smaller Δ*E*_ST_. This comparison also demonstrates that Δ*E*_ST_ is not a stand-alone predictor of TADF. A small Δ*E*_ST_ can facilitate reverse intersystem crossing, but the observed mechanism additionally depends on spin–orbit and vibronic coupling, state character, conformational dynamics, and the competition between radiative and non-radiative pathways.^10,52^ [PhPXZDBFDP]_2_Cu_4_I_4_ is more than 99% phosphorescent at ambient temperature despite Δ*E*_ST_ = 0.02 eV, whereas the PXZ analogue exhibits a substantial TADF contribution with Δ*E*_ST_ = 0.04 eV.^10^Fig. 3b therefore shows these observations without fitting a Δ*E*_ST_–*Φ*_PL_ correlation. The term “band gap” should also be used cautiously for discrete coordination clusters. The electrochemically estimated HOMO–LUMO gap, optical excitation energy, lowest singlet or triplet excitation, and Δ*E*_ST_ describe different quantities and should not be treated interchangeably. In the present dataset, the most defensible conclusion is that ligand substitution changes orbital localization and excited-state ordering; a general quantitative relationship between the HOMO–LUMO gap and *Φ*_PL_ cannot yet be established.

### 4.3 Crystal packing and host-environment effects

Crystal packing can influence Cu(i) cluster emission by modifying intermolecular contacts, conformational freedom, Cu⋯Cu and Cu–I geometries, exciton migration, and access to non-radiative decay pathways. These effects are most convincingly demonstrated using polymorphs or host–guest variants of the same compound, where molecular composition is retained while the local environment changes. Polymorphic Cu(i) iodide clusters provide direct evidence that relatively small packing-induced structural differences can alter emission color and stimulus responsiveness.^47^ More recent solvent-mediated crystal engineering of [Cu_4_I_4_(4-dpda)_4_] generated three compositionally related crystalline forms with distinct structures, room-temperature phosphorescence, high-energy/low-energy transfer barriers, and thermal-quenching behavior.^72^ These matched comparisons demonstrate that packing is an active photophysical variable. They do not, however, establish that dense packing universally enhances luminescence or that loosely packed structures necessarily undergo aggregation-caused quenching. Host dilution likewise modifies the photophysical behavior of Cu_4_I_4_ emitters. For [PXZDBFDP]_2_Cu_4_I_4_, changing from a neat film to a 20 wt% BCPO film shifts the emission maximum from 520 to 508 nm and reduces intermolecular interactions between adjacent cluster molecules.^10^ For the PhPXZ analogue, host dilution changes the relative contributions of the higher-energy charge-transfer emission and the lower-energy cluster-centered tail. These observations show that *λ*_em_, *Φ*_PL_, and *τ* obtained from crystals, powders, neat films, and doped host matrices cannot be considered directly interchangeable. Guest molecules may also alter emission through specific host–guest interactions rather than through a nonspecific packing effect. CuIDPO contains one-dimensional channels and a non-cuprophilic Cu⋯Cu separation of 3.38 Å. Exposure to CO_2_ produces pronounced luminescence enhancement, attributed to guest–host π–π interactions that restrict phenyl-group rotation.^33^ This result supports a reduction in non-radiative motion as the operative mechanism while also showing that strong cuprophilic interactions are not a prerequisite for TADF-related behavior in every Cu(i) cluster. Accordingly, statements that a particular packing arrangement “prevents ACQ” should be replaced by more conditional language. Restrictive packing, host dilution, or guest-induced conformational locking may suppress specific non-radiative or concentration-dependent pathways, but the outcome depends on the emitting state, intermolecular electronic coupling, defects, solvent content, and cluster concentration. Reliable packing–property correlations therefore require matched materials studied using SCXRD together with steady-state, time-resolved, and temperature-dependent spectroscopy.

### 4.4 Reconciling conflicting trends and establishing an evidence hierarchy

The apparently conflicting structure–property trends reported for Cu(i) coordination clusters largely arise because nuclearity, Cu⋯Cu separation, coordination geometry, ligand electronics, excited-state character, and crystal packing are rarely varied independently. Cross-comparison of chemically unrelated compounds can therefore generate correlations that disappear when the variables are examined in controlled series. Three levels of evidence can be distinguished. First, ligand-matched, polymorph-matched, or host-concentration-controlled comparisons provide the strongest basis for mechanistic interpretation. The DBFDP ligand series demonstrates that donor substitution can suppress low-lying cluster-centered contributions and improve *Φ*_PL_ without changing nuclearity.^88^ The PXZ/PhPXZ comparison shows that excited-state ordering and state character can outweigh the magnitude of Δ*E*_ST_.^10^ Polymorphic and solvent-engineered Cu_4_I_4_ systems demonstrate that packing can modify emission and thermal response while retaining the molecular composition.^47,72^ Second, correlations derived from related but not fully matched compounds should be regarded as conditional. Cu⋯Cu separation can influence cluster-centered state energies within a given structural family, but it cannot be used as a universal predictor of *Φ*_PL_. CuIDPO, for example, exhibits TADF-related behavior despite a Cu⋯Cu distance of 3.38 Å and the absence of a significant cuprophilic interaction.^33^ Likewise, ligand rigidity can reduce excited-state reorganization, but its effect may be overridden by state ordering, energy-gap-law behavior, or intermolecular quenching. Third, relationships inferred from broad compilations of different nuclearities, ligands, halides, and sample forms have low predictive strength. Such datasets are useful for identifying hypotheses but should not be presented as quantitative design laws. In particular, the present literature does not establish a universal monotonic nuclearity *λ*_em_ relationship, an inverse nuclearity *Φ*_PL_ relationship, or numerical Δ*θ* thresholds separating efficient and inefficient emitters. The available evidence instead supports a multivariable design framework. Ligand electronics primarily control orbital localization and the relative energies of ligand-centered, charge-transfer, and cluster-centered states. Structural constraint and packing influence excited-state reorganization and (*k*_{nr}_). Spin–orbit and vibronic coupling, together with Δ*E*_ST_ and the nature of the participating states, govern ISC/RISC competition. The measured *λ*_em_, *Φ*_PL_, and *τ* emerge from the combined action of these factors, as summarized in Fig. 3d. The evidential strength, appropriate interpretation, and principal limitations of the commonly employed structural descriptors are summarized in Table 3.

**Table 3:** Critical assessment of key structural descriptors governing the photophysical properties of luminescent Cu(i) coordination clusters, including their evidence strength and limitations. CC, cluster-centered; CT, charge transfer; RISC, reverse intersystem crossing; TADF, thermally activated delayed fluorescence

Structural descriptor	Experimentally supported relationship	Important limitation or counterexample	Evidence strength	Ref.
Nuclearity	Nuclearity can modify cluster-centered orbital delocalization and the number of accessible excited states	No universal monotonic relationship with *λ*_em_ or *Φ*_PL_ is established. Isonuclear Cu_4_I_4_ clusters exhibit *λ*_em_ ≈ 480–626 nm and *Φ*_PL_ ≈ 5–90%, depending mainly on ligand and environment	Low when considered alone	10 and 88
Cu⋯Cu separation	Changes in Cu⋯Cu distances within the same compound or polymorphic family can alter CC-state energy and emission color	Cu⋯Cu distances from chemically unrelated clusters cannot be directly correlated with *Φ*_PL_. CuIDPO exhibits TADF despite a non-cuprophilic Cu⋯Cu distance of 3.38 Å	Moderate for matched systems	33 and 47
Ligand rigidity	Rigid chelating ligands generally restrict excited-state structural reorganization and suppress non-radiative decay	Rigidity is not represented by a universal numerical Δ*θ* threshold; ligand electronics and packing may override geometric effects	Moderate, primarily qualitative	10,52,88
Ligand electronics	Donor substitution can change CT-state energies, suppress low-lying ^3^CC states and enhance radiative ligand-centred emission	Strong donor character does not guarantee TADF unless suitable S_1_/T_1_ coupling and excited-state ordering are also present	High within isostructural series	10 and 88
Singlet–triplet gap	A sufficiently small Δ*E*_ST_ can facilitate thermally activated RISC.	Small Δ*E*_ST_ is necessary but not sufficient: [PhPXZDBFDP]_2_Cu_4_I_4_ has Δ*E*_ST_ = 0.02 eV but remains >99% phosphorescent, whereas the PXZ analogue with Δ*E*_ST_ = 0.04 eV exhibits approximately 52% TADF.	High; experimentally demonstrated	10
Packing and host environment	Restricted intermolecular motion and suppression of concentration quenching can increase *Φ*_PL_ and modify *λ*_em_ and *τ*	Values measured in crystals, neat films, doped hosts or guest-loaded materials are not directly interchangeable	High only within matched material systems	10,49,72

Future studies should prioritize systematic series in which only one structural variable is changed at a time. Essential characterization should include SCXRD at relevant temperatures, absolute *Φ*_PL_, complete emission spectra, multiexponential decay analysis, temperature-dependent lifetimes and spectra, prompt/delayed spectral comparison, and quantum-chemical calculations of relaxed excited-state geometries and spin–vibronic coupling. Reporting the sample form, excitation wavelength, atmosphere, temperature, host identity, and dopant concentration is equally important.^77^ Until such standardized datasets become available, the most reliable design principles for Cu(i) clusters will remain those derived from matched molecular or polymorphic families rather than from nuclearity alone.

## 5. Transformation of Cu(i) clusters into hybrid functional materials

The practical deployment of discrete luminescent Cu(i) clusters requires their integration into processable, environmentally stable host matrices. Hybridization addresses the intrinsic limitations of discrete clusters poor solubility, oxidation susceptibility, and ACQ (with radioluminescent Cu(i) iodide ionic-chain hybrids illustrating this transformation,^9^ and biodegradable Cu(i) iodide cluster nanoparticles further demonstrating biomedical utility under low-dose X-ray irradiation^81^) while introducing synergistic functional properties unavailable in the pure cluster.^16,41,44^

### 5.1 Cluster-to-polymer and MOF-like hybrids

Covalent incorporation of Cu(i) clusters into polymer matrices significantly enhances resistance to air and moisture while preserving luminescence properties.^16^ Cluster-based MOF architectures have been designed as platforms for dynamic luminescence switching, with structural transformations induced by guest species enabling advanced anticounterfeiting applications.^15^ Chiral Cu(i) iodide cluster MOF hybrids exhibit highly efficient circularly polarized luminescence (*C*_PL_) with large dissymmetry factors (glum up to 0.1), opening avenues for chiroptical applications in 3D display and spintronics technologies,^13,38,63,79^ with related chiral copper(i) halide clusters and coordination chain polymers further expanding this structural family.^29^

### 5.2 Hybridization with oxides and carbon matrices

Integration of Cu(i) clusters with inorganic oxide supports (TiO_2_, ZnO, SiO_2_) confers thermal and photochemical stability, while carbon-based scaffolds (graphene derivatives, carbon dots, mesoporous carbon) facilitate efficient charge transport and separation. The sonochemical approach is particularly advantageous for these hybridizations: acoustic cavitation enables one-pot deposition or encapsulation of Cu(i) clusters onto oxide surfaces with superior dispersion and minimal Cu(i) oxidation.^17,19,21^ Cu NC@silica hybrids prepared by sonochemical routes exhibit enhanced photostability and emission brightness for sensing and bioimaging applications.^31^ Complementary Cu-cluster-based coordination polymers and MOF/COF platforms have likewise been used for volatile-organic-compound and mycotoxin sensing *via* Cu⋯Cu-interaction-modulated luminescence.^73,74^ Carbon based hybrids facilitate efficient photogenerated charge separation, enhancing visible-light-driven photocatalytic activity while spatially isolating Cu(i) clusters to prevent ACQ.^43,44,46^ The bifunctionality of such composites combining luminescent reporting with photocatalytic activity enables the development of self-monitoring catalytic systems, as discussed in Section 6.3.

### 5.3 Post-synthetic modification and defect engineering

Ligand exchange, surface encapsulation, and defect engineering offer additional routes to functional hybridization. Oxygen vacancies at Cu cluster oxide interfaces (*e.g.*, TiO_2−*x*_, ZnO_1−*x*_) enhance interfacial electronic coupling, improving both *Φ*_PL_ and photocatalytic activity through modulation of charge-transfer kinetics.^21,42^ Building on recent advances in oxide materials and sonochemistry, systematic investigation of defect–rich interfaces represents a promising direction for future research (Fig. 4).

**Fig. 4:**
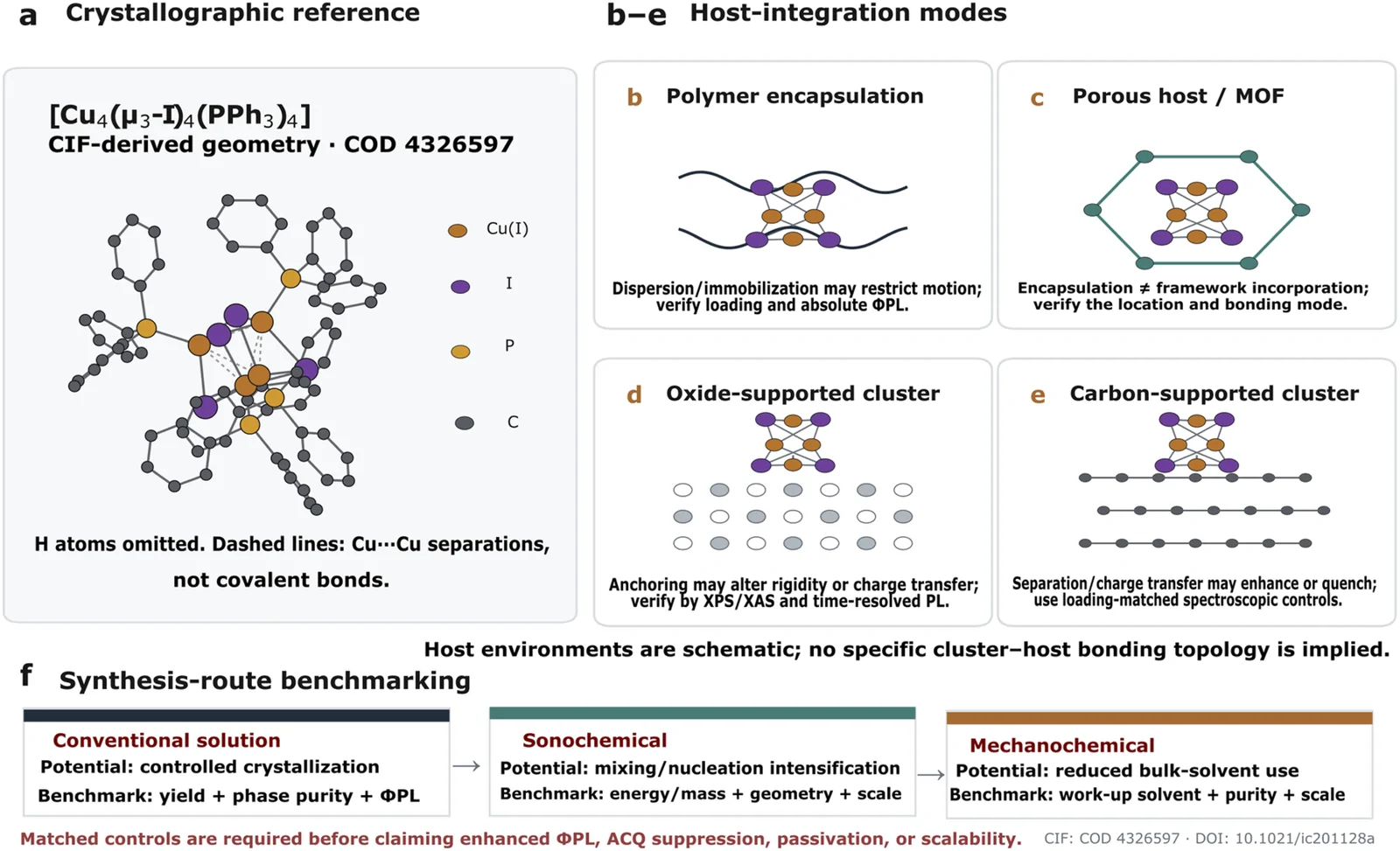
Structure-to-host integration and synthesis benchmarking: (a) CIF-derived structure of [Cu_4_(µ_3_-I)_4_(PPh_3_)_4_] (COD 4326597); (b–e) conceptual polymer, porous-host/MOF, oxide and carbon integration modes; and (f) criteria for matched comparison of conventional, sonochemical and mechanochemical routes. Host schematics do not imply specific cluster–host bonding topologies.

### 5.4 Critical assessment of hybridization strategies

Hybridization consistently improves environmental and photochemical stability, yet this benefit is frequently accompanied by partial luminescence quenching at the cluster–matrix interface, and the magnitude of this trade-off is rarely quantified on a common basis across the polymer, MOF, oxide, and carbon platforms discussed above. Furthermore, most sonochemical hybridization protocols reported to date have been demonstrated only at laboratory (sub-gram) scale, and long-term (multi-month) photostability data under operating conditions relevant to devices or sensors remain scarce. Systematic, side-by-side benchmarking of *Φ*_PL_ retention and stability across hybrid platforms is a clear gap for future work; our group's parallel experience with transition-metal MOF platforms for heavy-metal capture further underscores the broader versatility, and comparability challenges, of MOF-based functional materials beyond luminescence.^40^

## 6. Emerging applications

### 6.1 Chemical sensing and bioimaging

The stimuli-sensitive luminescence of Cu(i) clusters renders them attractive for sensor applications. Emission intensity, wavelength, or lifetime can be modulated by analytes including heavy metal ions (Ag^+^, Hg^2+^, Cu^2+^), volatile organic compounds (VOCs), temperature, pressure, and pH.^14,30,31,61,66,67^ Sonochemically synthesized Cu nanoclusters emitting in the red spectral region have demonstrated excellent sensitivity and selectivity for Ag^+^ detection in aqueous solution, with limits of detection in the nanomolar range.^14^ Related copper(i) iodide cluster and hybrid halide scintillators and inks, exhibiting near-unity *Φ*_PL_ and high-efficiency X-ray/CT-imaging response, further extend this application space.^83–87^ Hybrid composites of Cu(i) clusters with silica or carbon dots enhance aqueous dispersibility and biocompatibility for intracellular sensing and bioimaging.^31,58^ The mechanochromic and thermochromic behaviors of Cu(i) clusters arising from reversible changes in Cu⋯Cu distances and crystal packing further enable their use in optical security inks and anticounterfeiting materials,^3,15,36^ building on the broader family of mechanochromic copper iodide cluster compounds reviewed by Perruchas and co-workers.^71^

### 6.2 TADF-based optoelectronics

TADF in Cu(i) clusters has attracted intense interest for energy-efficient OLEDs and light-emitting electrochemical cells (LECs). The small Δ*E*_ST_ (0.1–0.2 eV) and microsecond-scale *τ* enable efficient harvesting of both singlet and triplet excitons, approaching 100% internal quantum efficiency.^11,12^ The excited-state reversal phenomenon in Cu_4_I_4_–PXZ clusters, as discussed in Section 3.1, has yielded prototype devices with *Φ*_PL_ ∼98% and EQE > 35%, representing a state-of-the-art benchmark for earth-abundant TADF emitters.^10^ Chiral Cu(i) cluster hybrids additionally enable circularly polarized electroluminescence, opening avenues for 3D display technologies and spin-selective optoelectronics.^38^ Hybridization with polymer or MOF matrices improves film-forming ability and operational stability, addressing a primary bottleneck for practical device integration.^16^

### 6.3 Photocatalysis and light-driven processes

Cu(i) clusters and their oxide/carbon hybrids are increasingly studied for visible-light photocatalysis. High nuclearity hydride clusters (Cu_20_, Cu_22_) show potential for hydrogenation and CO_2_ reduction reactions, leveraging their delocalized electron density and accessible metal hydride sites.^23,64,78^ Sonochemical cluster oxide carbon hybrids demonstrate enhanced charge separation for photocatalytic hydrogen evolution and pollutant degradation.^43,44,46^ The combination of photoluminescence with photocatalytic activity creates opportunities for “self-monitoring” systems in which emission intensity provides a real-time readout of catalyst performance or degradation state.^21,41^ While still less mature than noble-metal photocatalysis, the low cost, visible-light absorption, and tunable band gaps of Cu(i) clusters make them attractive for sustainable applications (Fig. 5).

**Fig. 5:**
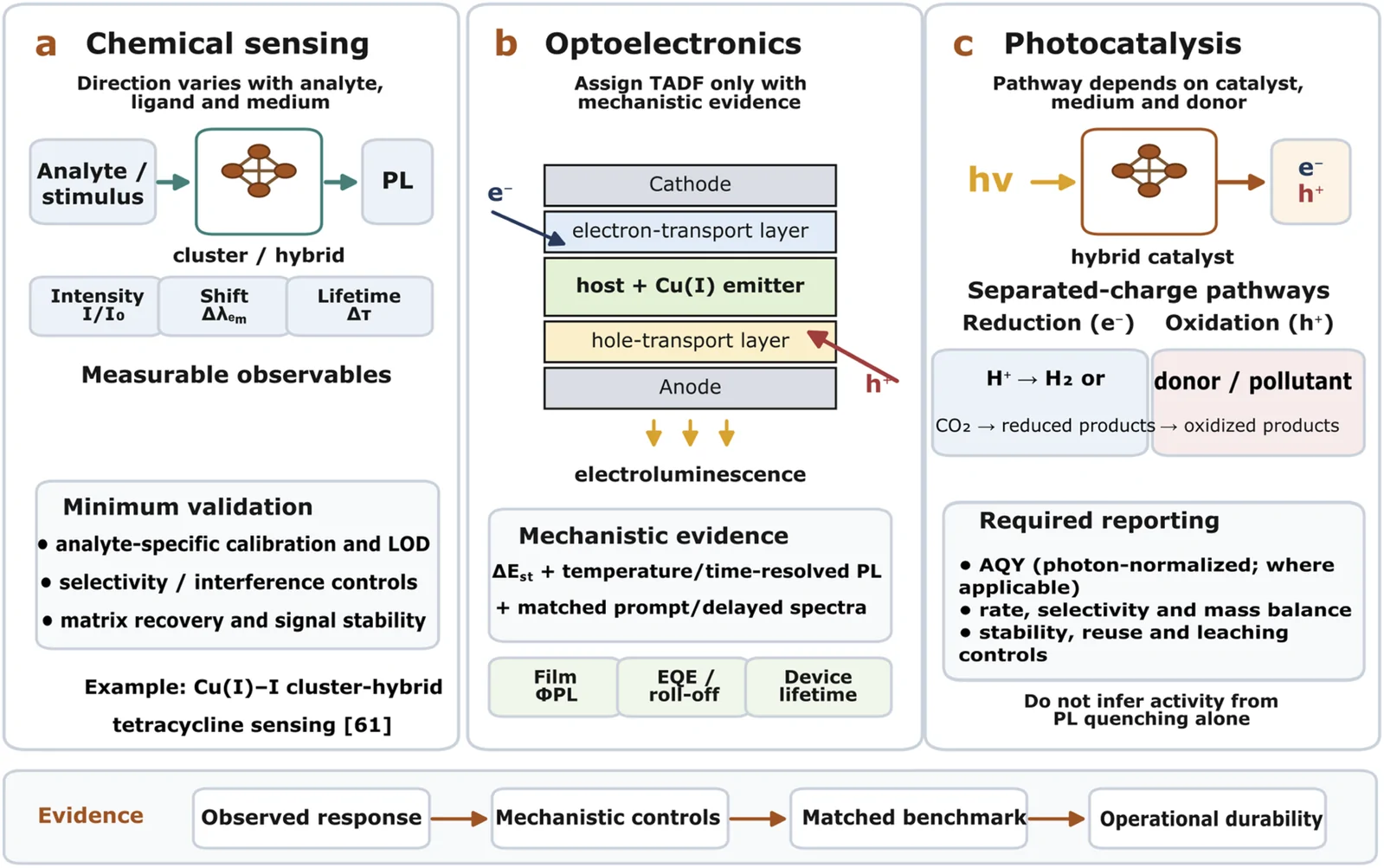
Evidence-based application framework for luminescent Cu(i) clusters and related hybrids: (a) system-dependent sensing, (b) optoelectronic integration and TADF validation, and (c) photocatalytic charge separation and reaction-specific benchmarking. Cluster icons are conceptual and do not represent a specific composition or topology.

### 6.4 Critical assessment of application readiness

Among the applications surveyed, TADF-based optoelectronics have received the most rigorous benchmarking (EQE, device lifetime under continuous operation), whereas sensing and photocatalysis studies more often report proof-of-concept performance (limit of detection, initial photocatalytic rate) without long-term operational or reusability data. Apparent quantum yields for photocatalytic hydrogen evolution and CO_2_ reduction are also rarely reported on a standardized actinometric basis, which complicates direct comparison with non-copper photocatalysts. Bridging this benchmarking gap is likely to be as important for the field's advancement as further gains in *Φ*_PL_ or Δ*θ* engineering.

## 7. Challenges, opportunities, and future perspectives

### 7.1 Current challenges

Despite significant advances, several fundamental challenges impede the practical deployment of Cu(i) coordination clusters. The primary challenge remains the inherent susceptibility of Cu(i) to oxidation to the photophysically silent Cu(ii) state, particularly in solution or under ambient conditions.^16,17^ ACQ and excited-state geometric distortion (Δ*θ*) continue to limit *Φ*_PL_ and long-term stability in many systems. Scalability represents a second major bottleneck: high-performance clusters are predominantly synthesized by small-scale solvothermal methods with poor batch-to-batch reproducibility and significant energy consumption.^17,44^ Additionally, while structure property relationships are increasingly well understood within specific ligand families, predictive design across diverse coordination environments particularly for achieving *Φ*_PL_ > 90% with operational stability remains challenging.^4,33^

### 7.2 Opportunities and emerging directions

A clear set of strategic opportunities has emerged from recent work:

• Scalable sonochemical synthesis: optimized ultrasound-assisted protocols for Cu_8_–Cu_22_ clusters and direct *in situ* hybridization with oxide or MOF matrices offer rapid reaction times, improved reproducibility, and reduced oxidation risk.^17,19,21,44^

• Defect-engineered hybrid interfaces: systematic introduction of oxygen vacancies on TiO_2_, ZnO, and CeO_2_ surfaces can modulate interfacial charge transfer, improving both *Φ*_PL_ and photocatalytic activity. Time-resolved spectroscopy combined with SCXRD will enable mechanistic understanding of these interfaces.^21,42^

• Multifunctional cluster–polymer/MOF composites: processable thin films and coatings incorporating Cu(i) clusters as dual luminophore–photocatalyst elements enable self-monitoring catalytic systems and scalable device fabrication.^16,44,46^

• Advanced structure–property mapping: high-throughput SCXRD combined with machine-learning-assisted DFT will enable comprehensive predictive models linking nuclearity, ligand rigidity, Cu⋯Cu distances, and packing motifs to photophysical outcomes.^4,10,48,60,88^

The convergence of these directions anchored by the structural diversity and TADF capability of Cu(i) clusters and the scalability of sonochemical synthesis positions this field as one of the most strategically important areas in contemporary coordination chemistry.

## 8. Conclusions

Luminescent Cu(i) coordination clusters have evolved from laboratory curiosities into a vibrant and practically significant class of functional materials. Through systematic control of nuclearity, ligand design, Cu⋯Cu interactions, and synthetic route, emission colors spanning the visible spectrum (480–720 nm) and quantum yields approaching unity (*Φ*_PL_ up to 99.9%) have been achieved in earth-abundant systems at costs and with structural diversities that compare favorably with, and in several respects extend beyond, those accessible to noble-metal analogues. The crystallographically grounded structure property framework established in this review relating Δ*θ*, Cu⋯Cu distances, HOMO–LUMO gaps, and intermolecular interactions to *Φ*_PL_ and *τ* provides a useful framework for rational design. The transformation of discrete clusters into polymer, MOF, oxide, and carbon-based hybrids, particularly *via* sonochemical routes, addresses the intrinsic stability and processability limitations of the molecular species while enabling synergistic optoelectronic and photocatalytic functionalities. Remaining challenges Cu(i) oxidation, ACQ, and synthetic scalability are tractable through defect engineering, surface passivation, and machine learning guided ligand design. Collectively, the earth-abundant composition, structural diversity, and TADF capability of Cu(i) coordination clusters establish them as highlights in the development of sustainable optoelectronic devices, sensing technologies, and visible light photocatalytic systems for the coming decade.

## Conflicts of interest

There are no conflicts of interest to declare.

## Data Availability

No new data were created or analyzed in this study. Data sharing is not applicable to this article as it is a review of existing literature and all discussed data are available in the cited references.
